# Clinical decision instruments for predicting mortality in patients with cirrhosis seeking emergency department care

**DOI:** 10.1111/acem.15088

**Published:** 2025-01-08

**Authors:** Swetha Parvataneni, Michelle Haugh, Yara Sarkis, Brittany Baker, Lauren D. Nephew, Marwan S. Ghabril, Raj Vuppalanchi, Eric S. Orman, Naga P. Chalasani, Archita P. Desai, Nicholas Eric Harrison

**Affiliations:** ^1^ Division of Gastroenterology and Hepatology Indiana University Indianapolis Indiana USA; ^2^ Department of Medicine Indiana University Indianapolis Indiana USA; ^3^ Department of Emergency Medicine Indiana University Indianapolis Indiana USA

**Keywords:** cirrhosis, clinical decision instrument, emergency department

## Abstract

**Objective:**

Clinical decision instruments (CDIs) could be useful to aid risk stratification and disposition of emergency department (ED) patients with cirrhosis. Our primary objective was to derive and internally validate a novel Cirrhosis Risk Instrument for Stratifying Post‐Emergency department mortality (CRISPE) for the outcomes of 14‐ and 30‐day post‐ED mortality. Secondarily, we externally validated the existing Model for End‐Stage Liver Disease (MELD) scores for explicit use in ED patients and prediction of the same outcomes.

**Methods:**

A cohort of 2093 adults with cirrhosis, at 16 sites in a statewide health system, was analyzed for 119 candidate variables available at ED disposition. LASSO with 10‐fold cross‐validation was used in variable selection for 14‐day (CRISPE‐14) and 30‐day (CRISPE‐30) logistic regression models. Area under the receiver operating characteristic curve (AUROC) was calculated for each variant of the CRISPE and MELD scores and compared via Delong's test. Predictions were compared to actual ED disposition for predictive value and reclassification statistics.

**Results:**

Median (interquartile range [IQR]) characteristics of the cohort were age 62 (53–70) years and MELD 3.0 13.0 (8.0–20.0). Mortality was 4.3% and 8.5% at 14 and 30 days, respectively. CRISPE‐14 and CRISPE‐30 outperformed each MELD variant, achieving AUROC of 0.824 (95% CI: 0.781–0.866) and 0.829 (0.796–0.861), respectively. MELD 3.0 AUROCs were 0.724 (0.667–0.781) and 0.715 (0.672–0.781), respectively. Compared to ED disposition, CRISPE‐14, CRISPE‐30, and MELD 3.0 significantly improved positive and negative predictive value and net reclassification index at multiple cutoffs. Applying CRISPE‐30 (cutoff 4.5) favorably reclassified one net ED disposition for mortality for every 12 patients, while MELD 3.0 net reclassified one disposition per 84 patients.

**Conclusions:**

CDIs may be useful in risk‐stratifying ED patients with cirrhosis and aiding disposition decision making. The novel CRISPE CDI showed powerful performance and requires external validation, while the existing MELD 3.0 score has moderate performance and is now externally‐validated in an ED population for short‐term mortality.

## INTRODUCTION

### Background

Emergency departments (ED) are the most common entry point for individuals seeking acute health care, including those with acute exacerbations of chronic conditions.^1^, ^2^ Individuals with cirrhosis account for 520,000 ED visits annually in the United States.^3^ They experience a one in three rate of ED revisits within 1 year, 71% of ED visits culminate in hospitalization, 31% experience readmissions within 30 days, and overall mortality reaches 15%.^4^, ^5^, ^6^, ^7^

### Importance

Clinical decision instruments (CDIs) may help ED clinicians optimize disposition and treatment decision making through improved risk stratification and are currently implemented for ED patients with numerous conditions (e.g., acute coronary syndrome, pulmonary embolus, blunt trauma, pneumonia, subarachnoid hemorrhage, many more^8^, ^9^, ^10^, ^11^, ^12^, ^13^, ^14^). High short‐term mortality and extensive health care utilization (HCU) associated with cirrhotic patients seeking ED care suggest that a CDI with strong performance characteristics could be useful in this population as well.

CDIs, and principally the Model for End‐Stage Liver Disease (MELD) scores, are validated to aid physician risk stratification of cirrhosis patients in the inpatient, hepatology outpatient, and perioperative settings. The original MELD score and its more recent iterations, MELD‐Na and MELD 3.0, are mortality prediction scores currently used to prioritize individuals awaiting liver transplantation and/or assess perioperative mortality in these non‐ED settings.^15^, ^16^, ^17^, ^18^ MELD/MELD‐Na/MELD 3.0 have not been validated specifically for short‐term (14‐ to 30‐day) mortality prediction or use specifically in ED patients, nor are any other CDIs validated for these purposes to our knowledge.

### Goals of this investigation

Our primary objective was to develop and internally validate a novel CDI for 30‐ and 14‐day mortality in ED patients with cirrhosis seeking care for all‐cause visits, the Cirrhosis Risk Instrument for Stratifying Post–Emergency department mortality (CRISPE). Our secondary objective was to externally validate the existing MELD score variants’ performance in an ED population, for prediction of the same ED‐relevant outcomes at the time of ED disposition.

## METHODS

### Study design and inclusion criteria

**FIGURE 1:**
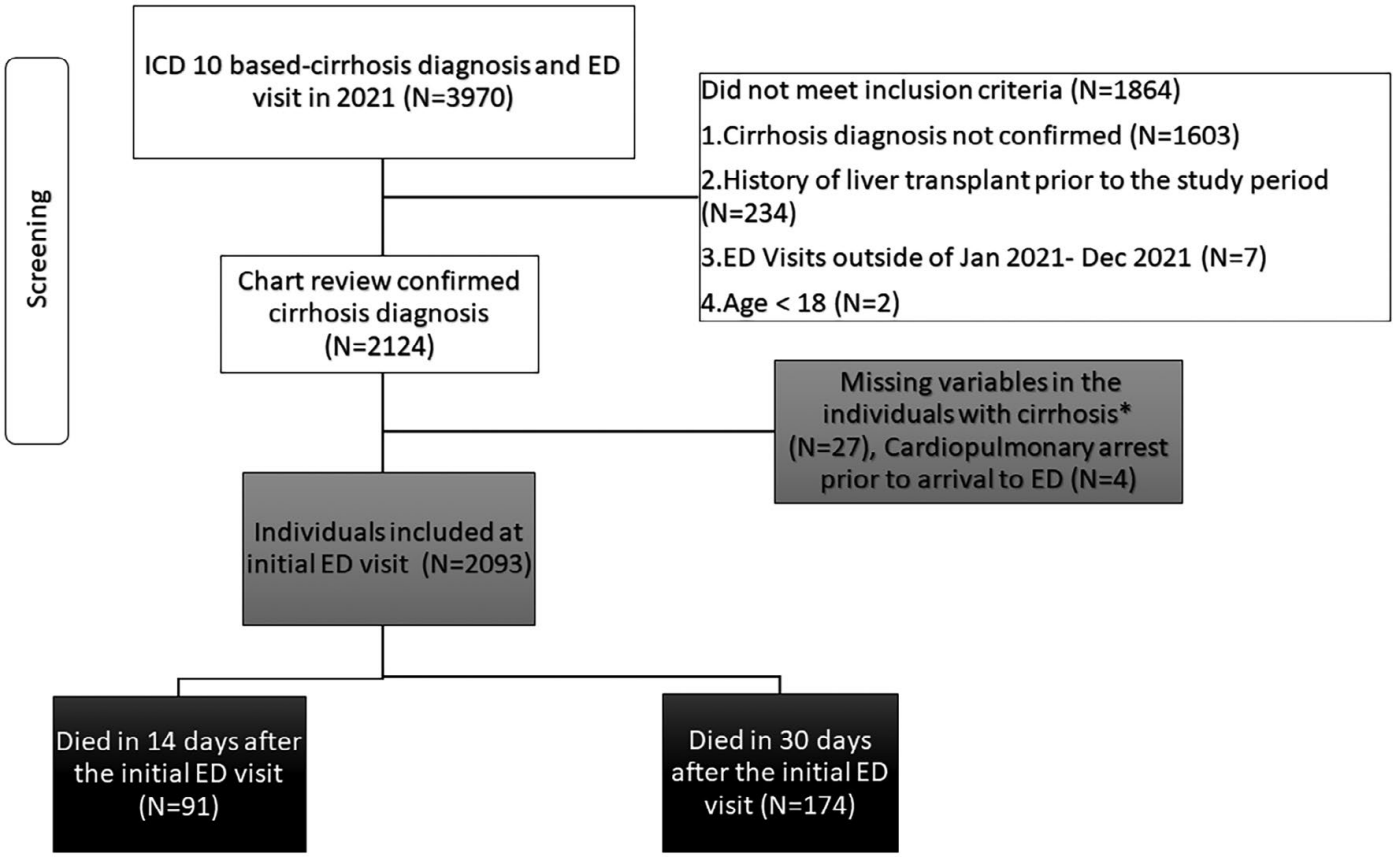
Inclusion diagram. Inclusion and exclusion of the cohort, which included 2093 patients with cirrhosis presenting to any of 16 different emergency departments (EDs). *Missingness of one or more vital signs was both uncommon after applying the other inclusion/exclusion criteria (*n* = 27/2124, 1.3%), and felt to be MAR since standard ED practice in each hospital was to obtain a full set of vital signs on every adult patient. MAR, missing at random.

We analyzed a registry of ED encounters for adult patients with cirrhosis (Figure 1), a description of which has been published previously.^19^ Briefly, the registry included all encounters at 16 EDs (one academic ED, 15 community EDs) for adult (≥18 years old) patients with cirrhosis. ED type ranged from a single 80,000‐visit‐per‐year large Level I trauma and liver transplant center to multiple small (<15,000 annual visit) critical access or free‐standing EDs. The registry was established via a statewide clinical data warehouse, with encounters screened by ICD‐10 codes for cirrhosis (Table S1) and data extraction technique described previously.^19^

For the current analysis, three categories of patients included in the main registry were excluded for considerations specific to risk stratification and disposition decision making (Figure 1). Patients with prior liver transplant were excluded from CDI development and/or validation because they have different ED considerations and physiology than those with cirrhosis and a native liver. Prehospital cardiopulmonary arrest was excluded to avoid biasing models with patients whom a clinician would obviously recognize dispositional needs and risk (i.e., universally, all need admission to an intensive care unit if they are able to be restored to spontaneous circulation in the ED). Finally, 1.3% (*n* = 27/2124) of patients were excluded for not having a full set of recorded ED vital signs, including respiratory rate (RR), heart rate, systolic blood pressure/diastolic blood pressure (DBP)/mean arterial blood pressures, oxygen saturation (SpO_2_), and temperature. Patients missing one or more vital signs were excluded under the assumption that obtaining a full set of vital signs during ED encounters with cirrhosis patients is a standard of care practice. Moreover, since this is indeed the standard of care at the 16 EDs in the analysis, encounters with a missing vital sign were hypothesized to be missing not at random (MNAR).

To maximize flexibility of CDI application across a broad range of presentations for cirrhosis patients, encounters for the current analysis were not restricted by reason for visit (e.g., gastrointestinal [GI] bleeding, sepsis, respiratory failure, sent to ED by outpatient provider for abnormal labs, etc.; see Table S1). Instead, all‐cause visits were considered, so long as the patient had a diagnosis of cirrhosis known prior to the ED encounter or newly diagnosed in the ED. One or more hepatology investigators manually reviewed charts to validate diagnosis of cirrhosis, adjudicate the encounter‐specific reason(s) for each ED visit, and validate other key variables (Table S1). Reviewers were blinded to the analysis, including variable selection and model fitting.

### Reporting of the study objectives

This report was prepared in accordance with the Transparent Reporting of a multivariable prediction model for Individual Prognosis or Diagnosis (TRIPOD) guidelines.^20^ The TRIPOD derivation and validation checklist^21^ was utilized for derivation and internal validation of CRISPE and is presented in Table S2. The TRIPOD validation checklist^21^, ^22^ was utilized for the external validation of MELD (Table S3).

### Data variables

A dataset of 119 variables (Table S1) available at the time of ED disposition, including vital signs, demographics, labs, imaging, social determinants of health, medical history, procedures, and hospital/posthospital course was assembled for eligible patients. The primary and secondary outcomes were 30‐ and 14‐day mortality, obtained from the Social Security Death Index.

Certain laboratory variables of interest were not obtained by the ED clinician during every encounter. When a given lab was not obtained prior to ED disposition and this lab was an input variable in one of the CDIs (CRISPE, MELD), we used a standardized normal value within the reference range (creatinine 0.9 mEq/L, sodium 140 mEq/L, International Normalized Ratio (INR) 1.0, albumin 4.0 g/dL, total bilirubin 0.5 mg/dL). This approach was chosen to align with practices utilized in the development of prior ED risk stratification tools, to allow clinical flexibility (i.e., allow use of the CDI when a certain lab was not obtained), and avoid biasing the model by missingness not at random (MNAR). First, using lab values obtained before ED arrival or after ED disposition (including during hospitalization) would reduce validity for use of the CDI specifically in ED patients. Second, we suspected that missing data procedures like multiple imputation would not be valid here since the decision to order or not order a given test likely has strong correlation with reason for ED presentation, ED providers’ usual care decision making, illness severity of the patient, and ultimately the outcomes of interest (i.e., mortality). As such, laboratory variables are potentially MAR and therefore not appropriate for multiple imputation. Third, use of a default “normal” value in the case of a lab not being obtained is consistent with derivation practices in other ED‐based CDIs (e.g., handling of troponin in derivation and validation of the Canadian Syncope Risk Score^23^, ^24^), which subsequently validated with strong performance^23^ (see the supplement for further references and details regarding this strategy and rates of missingness by laboratory variable). A sensitivity analysis was performed to test area under the receiver operating characteristic curve (AUROC) in only those patients with complete laboratory data (Data S1).

### Statistical analyses

#### Derivation and internal validation of CRISPE

A novel CDI for post‐ED mortality, CRISPE, was derived and internally validated. Variables for logistic regression models (30‐day [CRISPE‐30] and 14‐day [CRISPE‐14] mortality) were selected initially via LASSO with 10‐fold cross‐validation for model AUROC. To enhance model parsimony, selection was further limited to a maximum 10 events per variable. Categorical variables with <10% prevalence were excluded. For CRISPE‐30, histories of solid tumor and hepatocellular carcinoma (HCC) had substantial overlap and were combined.

Variables were transformed where appropriate with restricted cubic splines, provided that analysis of variance of the linear versus nonlinear terms showed *p* < 0.05 for the latter. Cross‐validated model calibration was performed to ensure mean absolute error of ≤0.5%. One variable, temperature, was dropped due to adverse effects on calibration. Bias estimation by the bootstrap‐based method described by Harrell^25^ was used to penalize optimism in model AUROC (i.e., giving a more conservative internal validation AUROC estimate compared to observed AUROC). An electronic medical record (EMR) version of each model was constructed by excluding variables within CRISPE‐30 and CRISPE‐14 requiring clinician judgment. After deriving the prediction model, we also performed sensitivity analysis by excluding individuals with cirrhosis who underwent liver transplants within 30 days.

For internal validation, we used the bootstrap approach of Harrell, instead of random splitting the data into derivation and validation cohorts.^20^, ^25^ The TRIPOD author group has noted that random splitting of the data, while sometimes thought of as “external validation,” is in fact an inefficient form of internal validation only.^20^ The bootstrap method is noted by the TRIPOD group to perform equivalently robust internal validation without the cost of these inefficiencies and has the added benefit of estimating the performance of the developed model adjusted for overfitting (“optimism”).

#### External validation of MELD variants

Each version of MELD (the original MELD score, MELD‐Na, MELD 3.0; in order of oldest to newest, respectively) was calculated using only those variables available prior to ED disposition to estimate real‐world performance in an ED population with data available to ED providers. MELD variants’ scores were calculated using the point values assigned for each variable as previously defined during development in non‐ED settings,^15^, ^16^, ^17^, ^18^ without adjustment. The inclusion and exclusion criteria, approach to missing values, and other data considerations were the same as those described above to facilitate a like‐to‐like comparison of models (i.e., MELD vs. CRISPE). MELD variant scores were compared to the same short‐term (30‐day and 14‐day) mortality outcomes, at varying cutoffs.

#### Model performance assessment and comparison

Receiver operating characteristic (ROC) curves were plotted for each version of CRISPE and compared by AUROC to MELD, MELD‐Na, and MELD 3.0 (DeLong's test). ED physician disposition (discharge vs. observation or admission) was used as a benchmark for usual care risk stratification. Test characteristics (sensitivity, specificity, negative predictive value [NPV], positive predictive value [PPV]) were calculated for ED disposition versus each outcome and compared to CRISPE‐14 and CRISPE‐30 at multiple cutoffs of predicted risk (McNemar's test). The continuous net reclassification index (NRI) of CDIs versus actual disposition was calculated at selected cutoffs. “Appropriate reclassifications” were defined as reclassified false positives (i.e., admissions among survivors reclassified by CRISPE to discharge) plus reclassified false negatives (i.e., ED discharges reclassified to admission among decedents). “Inappropriate reclassifications” were defined as reclassified true positives plus reclassified true negatives. Net appropriate reclassification rate was defined as appropriate minus inappropriate, divided by total patients. The number needed to diagnose (NND) to net‐reclassify one disposition appropriately was this rate's inverse.

## RESULTS

### Study population

Table 1 summarizes cohort characteristics (*N* = 2093). Median (interquartile range [IQR]) age was 62 (53–70) years, with 53% being male, 90% White, and 98% non‐Hispanic. Following the index ED visit, 8.3% (*n* = 174) died within 30 days and 4.3% (*n* = 91) within 14 days. ED disposition was discharge for 42% of patients (*n* = 881).

**TABLE 1:** Demographics and clinical characteristics of study cohort.

Characteristic	Total cohort (*N* = 2093)	30‐day survivors after the initial ED visit (*n* = 1919)	Died within 30 days after the initial ED visit (*n* = 174)	*p*‐value
Age (years)	62 (53–70)	62 (53–69)	66 (58–73)	<0.001
Male gender	1113 (53)	1013 (53)	100 (57)	0.20
Race				
White	1874 (90)	1717 (91)	157 (94)	0.60
Non‐White^a^	219 (10)	202 (9.5)	17 (6)	0.60
Non‐Hispanic ethnicity	2021 (98)	1854 (97)	167 (99)	0.40
Living situation prior to ED presentation				0.10
Home	1897 (91)	1745 (91)	152 (87)	0.12
Subacute rehab or skilled nursing facility	46 (2)	39 (2)	7 (4)	0.10
Other^b^	146 (7)	131 (7)	15 (9)	0.53
Elixhauser Comorbidity Index	7.0 (4.0–10.0)	7.0 (4.0–10.0)	6.0 (3.0–9.0)	0.12
Newly diagnosed with cirrhosis in the ED	390 (18.4)	348 (18)	36 (21)	0.40
Etiology of cirrhosis				
Alcohol	788 (38)	718 (37)	70 (40)	0.50
MASH	557 (27)	516 (27)	41 (24)	0.30
Viral hepatitis	440 (21)	414 (22)	26 (15)	0.04
Other^c^	190 (10)	179 (9)	11 (6)	0.17
Undetermined/unknown^c^	366 (18)	324 (17)	42 (24)	0.02
Complications of cirrhosis				
Ascites	1017 (49)	904 (47)	113 (65)	<0.001
Hepatic encephalopathy	667 (32)	594 (31)	73 (42)	0.003
History of varices	712 (34)	656 (34)	56 (32)	0.60
Variceal bleeding	211 (10)	194 (10)	17 (10)	0.90
TIPS	121 (6)	115 (6)	6 (3)	0.20
HCC	145 (7)	121 (6)	24 (14)	<0.001
Social Deprivation Index	55.0 (28.0–81.0)	55.0 (28–82)	49.0 (29–77)	0.50
Social drivers of ED visit				
None	1716 (82)	1567 (82)	149 (86)	0.20
Active alcohol use	257 (12)	240 (13)	17 (10)	0.30
Active other substance use	38 (1.8)	37 (1.9)	1 (0.6)	0.40
Nonadherence to medications or diet	79 (4)	75 (4)	4 (2)	0.30
Transport or caregiver Issues	18 (1)	15 (1)	3 (2)	0.29
Unable to get visit to PCP or specialist	36 (2)	35 (1)	1 (1)	0.38
Health care use in 12 months prior				
Prior ED encounters	0.00 (0.00–1.00)	0.00 (0.00–1.00)	0.00 (0.00–0.00)	0.013
Prior inpatient encounters	0.00 (0.00–1.00)	0.00 (0.00–1.00)	0.00 (0.00–1.00)	0.20
Prior outpatient encounters	2.00 (0.00–6.00)	2.00 (0.00–6.00)	1.00 (0.00–5.00)	0.30
No‐show visits to clinic	0.00 (0.00–0.00)	0.00 (0.00–0.00)	0.00 (0.00–0.00)	0.40
Established with GI/hepatology within the same health care system	1246 (60)	1162 (61)	84 (48)	0.002
Established with GI/hepatology outside	126 (6)	116 (6)	10 (6)	0.90

*Note*: Data are reported as median (IQR) or *n* (%).

Abbreviations: GI, gastrointestinal; HCC, hepatocellular carcinoma; MASH, metabolic associated steatohepatitis; PCP, primary care provider; TIPS, transjugular intrahepatic portosystemic shunt.

^a^ Other race categories include Black (8.26%), Asian (0.76%), American Indian or Alaskan Native (0.14%), and Native Hawaiian or Pacific Islander (0.05%).

^b^ Other living situation besides at home or at skilled nursing or subacute rehab facility, e.g., homeless, public shelter, or unknown.

^c^ Other etiologies of liver disease: autoimmune, cholestatic, cryptogenic, Budd–Chiari syndrome, hereditary hemochromatosis, alpha‐1 antitrypsin deficiency, congenital biliary atresia, IgG4 sclerosing cholangitis, congenital hepatic fibrosis, cystic fibrosis, granulomatous hepatitis, sarcoidosis.

Decedents at 30 days were significantly (*p* < 0.05) more likely to have older age (66 years vs. 62 years), undetermined cirrhosis etiology (24% vs. 17%), ascites (65% vs. 44%), hepatic encephalopathy (42% vs. 31%), HCC (14% vs. 6%), and fewer prior ED visits in the past year. They were less likely (*p* < 0.05) to have an established gastroenterologist/hepatologist (48% vs. 61%) or viral hepatitis (15% vs. 22%).

Table 2 summarizes ED visit characteristics. Decedents at 30 days were significantly (*p* < 0.05) more likely to visit the ED for shortness of breath (25% vs. 18%), altered mental status (23% vs. 10%), and fall/generalized weakness (20% vs. 13%) and be sent to the ED by their doctor (19% vs. 13%). They also received more ED testing including serum labs (97% vs. 91%), urinalysis (25% vs. 19%), blood cultures (14% vs. 6%), chest x‐ray, computed tomography (head, abdomen), and abdominal ultrasound (all *p* < 0.05). Decedents also had higher MELD variant scores based on ED labs (*p* < 0.001). A liver transplant was performed in three individuals within 30 days after the initial ED visit, and exclusion of these patients did not significantly affect any performance characteristics described below.

**TABLE 2:** Characteristics of initial ED visit.

Characteristic	Total cohort (*N* = 2093)	30‐day survivors after the initial ED visit (*n* = 1919)	Died within 30 days after the initial ED visit (*n* = 174)	*p*‐value
ED visit outside business hours	796 (38)	726 (38)	70 (40)	0.70
ED visit during weekend	487 (23)	453 (24)	34 (20)	0.20
Reason for visit				
Abdominal pain	434 (21)	401 (21)	33 (19)	0.50
Shortness of breath	400 (19)	357 (18)	43 (25)	0.003
Ascites/abdominal distension/edema/volume overload	338 (19)	355 (19)	31 (18)	>0.9
Fall/generalized weakness	291 (14)	256 (13)	35 (20)	0.013
Doctor instructions^a^	280 (14)	246 (13)	34 (19)	0.010
GI symptoms^b^	270 (13)	245 (13)	25 (14)	0.51
Altered mental status	236 (11)	196 (10)	40 (23)	<0.001
Musculoskeletal pain/swelling	179 (8)	172 (9)	7 (4)	0.026
GI bleeding	166 (8)	151 (8)	15 (9)	0.70
Chest pain	130 (6)	125 (7)	5 (3)	0.057
Interventions during visit^c^				
Lab	1906 (91)	1738 (91)	168 (97)	0.008
Urine studies	411 (20)	367 (19)	44 (25)	0.050
Blood cultures	146 (7)	121 (6)	25 (14)	<0.001
Imaging during visit^c^				
Chest x‐ray	797 (38)	714 (37)	83 (48)	0.006
CT abdomen	733 (35)	653 (34)	80 (46)	0.002
CT chest	277 (13)	247 (13)	30 (17)	0.10
CT head	386 (19)	343 (18)	43 (25)	0.026
Abdominal ultrasound	109 (5)	94 (5)	15 (9)	0.034
Procedures during the ED visit				
Paracentesis	83 (4)	73 (4)	10 (6)	0.20
Thoracentesis	3 (0.1)	3 (0.2)	0 (0)	>0.9
Intubation	6	2 (0.1)	4 (2)	<0.001
Central venous catheter	4 (0.2)	1 (<0.1)	3 (2)	0.002
ED consults				
GI/hepatology	129 (6)	117 (6)	12 (7)	0.80
Other^d^	63 (3)	55 (2.9)	8 (4.6)	0.20
Labs at the initial ED visit				
Sodium	137.0 (133.0–140.0)	137.0 (134.0–140.0)	134.0 (129.0–138.0)	<0.001
Bilirubin	1.00 (0.50–2.20)	0.90 (0.50–2.00)	1.95 (0.73–5.50)	<0.001
Albumin	3.60 (3.00–4.00)	3.70 (3.10–4.00)	2.90 (2.60–3.50)	<0.001
Creatinine	0.90 (0.77–1.23)	0.90 (0.76–1.20)	1.12 (0.86–1.83)	<0.001
INR	1.00 (1.00–1.32)	1.00 (1.00–1.30)	1.29 (1.00–1.64)	<0.001
MELD variants based on ED labs				
MELD^a^	10 (6–16)	10 (6–15)	16 (10–22)	<0.001
MELD‐Na^a^	10 (6–18)	9 (6–17)	18.0 (10–27)	<0.001
MELD 3.0^a^	13 (8–20)	12.0 (8–19)	22.0 (14–32)	<0.001
Disposition				
Hospitalized	1186 (57)	1039 (54)	147 (84)	<0.001
Home	881 (42)	854 (45)	27 (16)	<0.001
ED observation unit	26 (1)	26 (1.4)	0 (0)	0.20

*Note*: Data are reported as *n* (%) or median (IQR).

Abbreviations: GI, gastrointestinal; INR, International Normalized Ratio; IQR, interquartile range; MELD, Model for End‐Stage Liver Disease.

^a^ Doctors instructions to go to ED based on abnormal outpatient labs, imaging, vitals, or another reason.

^b^ GI symptoms: nausea, vomiting, diarrhea, other GI symptom such as dysphagia.

^c^ During first 8 h of ED visit.

^d^ Other: pulmonology, critical care, general surgery, nephrology, cardiology, neurology, oncology, gynecology, neurosurgery, hospitalist, interventional radiology, psychiatry, orthopedics, podiatry, ENT, urology, vascular, social work.

### The CRISPE tool for 30‐day and 14‐day mortality

For CRISPE‐30, 16 multivariable predictors were selected compared to eight for CRISPE‐14. The multivariable adjusted odds ratio (OR) for each model's variables are presented in Table 3. CRISPE‐14 included age, ED altered mental status, three ED labs (creatinine, bilirubin, albumin), and three ED vital signs (RR, DBP, and shock index). CRISPE‐30 included each of these in addition to active alcohol use history, ED dyspnea, established GI/hepatologist within the same health system as the ED, history of solid cancer, serum sodium, and uncontrolled ascites (defined as ascites with one or more large‐volume paracentesis procedures in previous 3 months and/or not currently treated with diuretics or transjugular intrahepatic portosystemic shunt [TIPS]). An online calculator for the final CRISPE‐30 and CRISPE‐14 tools is available at https://redcap.link/CRISPE.

**TABLE 3:** Multivariable‐adjusted predictors of 14‐ and 30‐day mortality in the CRISPE‐30 and CRISPE‐14 CDIs.

Predictor	30‐day mortality		14‐day mortality	
	Adjusted OR	95% CI	Adjusted OR	95% CI
Age	**2.21**	**1.67–2.94**	**2.52**	**1.77–3.60**
Active alcohol use	**0.52**	**0.27–0.98**	—	—
Current established outpatient GI/hepatology care in the same health system as the ED	**0.58**	**0.40–0.82**	—	—
Uncontrolled^a^ ascites at baseline	**1.47**	**1.00–2.16**	—	—
HCC or other solid tumor	**2.00**	**1.32–3.00**	—	—
Shortness of breath in ED	1.32	0.85–2.04	—	—
Altered mental status in ED	**1.90**	**1.21–2.98**	**2.02**	**1.18–3.24**
Creatinine	1.03	0.98–1.09	1.06	0.99–1.13
Sodium	**0.77**	**0.61–0.96**	—	—
Bilirubin	**1.18**	**1.11–1.24**	**1.16**	**1.09–1.24**
Albumin	**0.46**	**0.34–0.63**	**0.49**	**0.33–0.71**
SpO_2_	0.91	0.79–1.04	—	—
Shock index^b^	**1.37**	**1.13–1.67**	**1.54**	**1.21–1.96**

Nonlinear predictors^c^		Adjusted OR^c^	95% CI	Adjusted OR^c^	95% CI
Predictor^c^	Value of predictor^c^				
RR^c^	**4 vs. 20**	**4.59**	**0.97–21.8**	**9.56**	**1.53–59.9**
	**8 vs. 20**	**1.81**	**0.92–3.54**	**4.84**	**1.30–18.0**
	**12 vs. 20**	**1.57**	**1.02–2.43**	**2.45**	**1.11–5.42**
	16 vs. 20	1.15	0.90–1.47	1.26	0.95–1.69
	20 vs. 20	1.00	1.00–1.00	1.00	1.00–1.00
	**24 vs. 20**	**1.30**	**1.08–1.56**	**1.38**	**1.12–1.69**
	**28 vs. 20**	**1.81**	**1.21–2.69**	**2.09**	1.35–3.24
	**32 vs. 20**	**2.52**	**1.36–4.65**	**3.17**	**1.61–6.22**
	**36 vs. 20**	**3.51**	**1.53–8.04**	**4.80**	**1.93–12.0**
	**40 vs. 20**	**4.88**	**1.72–13.89**	**7.28**	**2.31–23.0**
DBP^c^	**20 vs. 80**	**3.95**	**1.40–11.1**	**4.15**	**1.19–14.5**
	**40 vs. 80**	**2.33**	1.23–4.41	**2.40**	**1.11–5.19**
	**60 vs. 80**	**1.37**	**1.06–1.77**	**1.39**	**1.02–1.89**
	80 vs. 80	1.00	1.00–1.00	1.00	1.00–1.00
	80 vs. 100	1.17	0.83–1.64	1.17	0.75–1.81
	80 vs. 120	1.47	0.71–3.05	1.47	0.57–3.82
	80 vs. 140	1.85	0.60–5.72	1.86	0.43–8.06
	80 vs. 160	2.33	0.51–10.7	2.35	0.32–17.0

*Note*: Multivariable‐adjusted ORs for continuous variables are scaled to a change equal to the width of the IQR, unless otherwise specified. Bold: statistically significant at *p* < 0.05.

Abbreviations: CRISPE‐14, Cirrhosis Risk Instrument for Stratifying Post‐Emergency department mortality 14‐day mortality model; CRISPE‐30, Cirrhosis Risk Instrument for Stratifying Post‐Emergency department mortality 30‐day mortality model; DBP, diastolic blood pressure; HCC, hepatocellular carcinoma; HR, heart rate; RR, respiratory rate; SpO_2_, oxygen saturation; TIPS, transjugular intrahepatic portosystemic shunt.

^a^ Defined as ascites with one of more large‐volume (i.e. therapeutic) paracentesis procedures in previous 3 months, and/or reccurent ascites not currently treated with diuretics or TIPS.

^b^ HR divided by systolic blood pressure.

^c^ Nonlinear term fit by restricted cubic spline, so OR varies with the value of the predictor. The adjusted OR is presented at various values of the predictor, compared to a “normal” value (20 for RR, 80 mm Hg for DBP); e.g., the adjusted OR of 4.59 for RR at “4 vs. 20” indicates that a respiratory rate of 4 is associated with 4.59‐fold greater odds of death than a rate of 20. In general terms, RR and DBP were associated with greater mortality at their extremes (very low and very high), and lower mortality at “normal” values.

### Performance of MELD, MELD‐Na, MELD 3.0, and CRISPE‐30 for predicting 30‐day mortality

**FIGURE 2:**
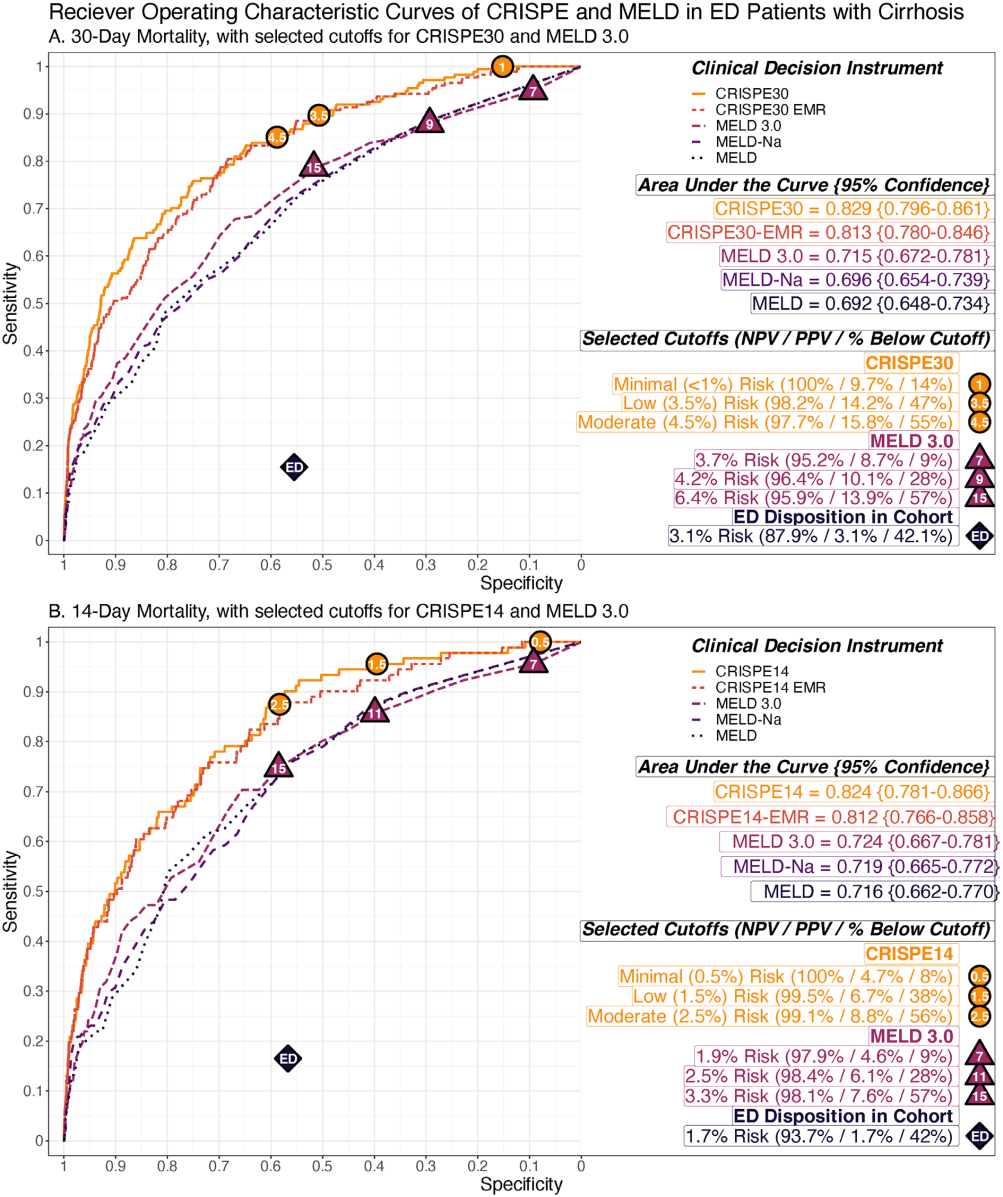
ROC curves with selected cutoffs for CRISPE‐30, CRISPE‐14, and MELD variants in ED patients with cirrhosis. ROC curves for (A) 30‐day (CRISPE‐30, CRISPE‐30‐EMR, MELD variants) and (B) 14‐day mortality models (CRISPE‐14, CRISPE‐14‐EMR, MELD variants). Selected cutoffs for CRISPE (orange circles) and MELD 3.0 (purple triangles) are plotted on the ROCs themselves. Labeling of cutoffs with NPV, PPV, and the percentage of the cohort above versus below the cutoff is included at the bottom right of each panel, underneath area under the ROC for each model. Sensitivity, specificity, and predictive values of usual care ED disposition decisions are included at the black diamond. For a detailed description and comparison of CRISPE, MELD, and ED disposition across multiple cutoffs by predictive values, sensitivity, specificity, net reclassification, and 2 × 2 tables, please refer to Table S2 in the supplement. CRISPE, Cirrhosis Risk Instrument for Stratifying Post‐Emergency department mortality; CRISPE‐14, CRISPE 14‐day mortality model; CRISPE‐30, CRISPE 30‐day mortality model; MELD, Model for End‐Stage Liver Disease; NPV, negative predictive value; PPV, positive predictive value; ROC, receiver operating characteristic.

Figure 2A presents ROCs for MELD, MELD‐Na, MELD 3.0, and CRISPE‐30. AUROC for 30‐day mortality was 0.715 for MELD 3.0 (0.672–0.781). When excluding cases where one or more laboratory values was not obtained by the physician, instead of assuming a standardized normal value, MELD 3.0 AUROC was significantly worse (0.646; see supplement for full results of sensitivity analysis). MELD 3.0 had significantly greater AUROC than original MELD (+0.023, 0.005–0.042) and MELD‐Na (+0.020, 0.002–0.035).

AUROC for CRISPE‐30 was 0.829 (0.796–0.861). CRISPE‐30 outperformed all three MELD variants (all *p* < 0.001), with a difference in AUROC compared to MELD 3.0 of +0.114 (0.080–0.148). After bias estimation and penalization, optimism‐corrected AUROC (i.e., expected performance in an external sample) was 0.816 for CRISPE‐30. CRISPE‐30 AUROC was similar (0.818) when excluding cases where one or more laboratory values was not obtained (sensitivity analysis, supplement), compared to assumption of a standardized normal lab value (Methods) when missing.

### Performance of MELD, MELD‐Na, MELD 3.0, and CRISPE‐14 for predicting 14‐day mortality

Figure 2B presents ROC curves of the MELD variants and CRISPE‐14. MELD 3.0 AUROC was 0.724 (0.667–0.781). When excluding cases where one or more laboratory values was not obtained by the physician, MELD 3.0 AUROC for 14‐day mortality was lower (0.692) though not significantly different (see supplement for full results of sensitivity analysis). Higher AUROC for MELD 3.0 versus original MELD and MELD‐Na for 14‐day mortality (*p* = 0.622, *p* = 0.381, respectively) was not statistically significant.

AUROC for CRISPE‐14 was 0.824 (0.781–0.866). CRISPE‐14 outperformed each MELD variant (all *p* < 0.001), with difference in AUROC of +0.100 (0.055–0.143) compared to MELD 3.0 (Figure 2). After bias penalization, optimism‐corrected AUROC was 0.810. CRISPE‐14 AUROC was similar (0.836) when excluding cases where one or more laboratory values was not obtained (sensitivity analysis, supplement), compared to assumption of a standardized normal lab value (Methods) when missing.

### CRISPE EMR‐integration models

CRISPE‐30‐EMR was created by removing potentially subjective variables to facilitate direct‐to‐EMR integration. CRISPE‐30‐EMR retained 10 variables (excluded altered mental status, active alcohol use, GI follow‐up, uncontrolled ascites, dyspnea) and CRISPE‐14‐EMR retained six variables (excluded altered mental status). AUROCs were 0.813 (0.780–0.846) and 0.803 (0.769–0.836), respectively. Both retained a statistically significant improvement in AUC compared to MELD 3.0 (Figure 2).

### Reclassification and predictive value compared to ED physician disposition

Table 4 and Figure 2 compare classification of mortality and predictive value by ED disposition versus CRISPE and MELD 3.0. ED physicians discharged 42.1% (*n* = 881) patients, in whom mortality was lower than admitted patients (*p* < 0.001) at 14 (1.7% vs. 6.3%) and 30 days (3.1% vs. 12.1%). NPVs of ED disposition were 93.7% (95% CI 92.2–95.0) and 87.9% (85.9–89.7), for predicting 14‐ and 30‐day mortality, respectively.

**TABLE 4:** Mortality classification and predictive value by ED disposition compared to CRISPE‐14, CRISPE‐30, and MELD 3.0.

Classifier	Outcome	Score cutoff^a^	Predicted probability of outcome	NRI^b^	NPV	PPV				
							Decedents^c^	Survivors^c^		
							TP	FN	FP	TN
ED disposition	14‐day death	Discharge	1.7% (1.0–2.8)	–	93.7% (92.2–95.0)	1.7% (1.0–2.8)	15	76	866	1136
CRISPE‐14	14‐day death	0.5	0.5% (0.3–0.8)	**0.35** (0.27–0.43) *	**100%** (97.7–100)*	**4.7%** (3.8–5.7)*	91	0	1844	158
CRISPE‐14	14‐day death	1	1% (0.7–1.5)	**0.51** (0.42–0.60) *	**99.6%** (98.7–100)*	**5.7%** (4.6–7.0)*	89	2	1471	531
CRISPE‐14	14‐day death	1.5	1.5% (1.0–2.1)	**0.62** (0.51–0.73) *	**99.5%** (98.7–99.9)*	**6.7%** (5.4–8.2)*	87	4	1212	790
CRISPE‐14	14‐day death	2	2% (1.4–2.8)	**0.70** (0.59–0.82) *	**99.4%** (98.7–99.8)*	**7.8%** (6.3–9.6)*	85	6	1004	998
CRISPE‐14	14‐day death	2.5	2.5% (1.9–3.4)	**0.73** (0.61–0.85) *	**99.1%** (98.3–99.5)*	**8.8%** (7.0–10.8)*	80	11	834	1168
CRISPE‐14	14‐day death	3	3% (2.3–3.9)	**0.72** (0.59–0.84) *	**98.6%** (97.8–99.2)*	**9.3%** (7.4–11.6)*	73	18	709	1293
CRISPE‐14	14‐day death	3.5	3.5% (2.7–4.5)	**0.74** (0.62–0.87) *	**98.6%** (97.8–99.1)*	**10.3%** (8.2–12.9)*	71	20	615	1387
CRISPE‐14	14‐day death	4	4% (3.1–5.1)	**0.73** (0.60–0.86) *	**98.3%** (97.5–98.9)*	**11.1%** (8.7–13.9)*	66	25	527	1475
CRISPE‐14	14‐day death	4.5	4.5% (3.5–5.7)	**0.71** (0.58–0.84) *	**98.1%** (97.3–98.7)*	**11.6%** (9.0–14.7)*	61	30	463	1539
CRISPE‐14	14‐day death	5	5% (4.0–6.2)	**0.72** (0.59–0.85) *	**98.1%** (97.3–98.7)*	**12.7%** (9.8–16.0)*	60	31	413	1589
CRISPE‐14	14‐day death	5.5	5.5% (4.4–6.8)	**0.74** (0.61–0.87) *	**98.1%** (97.4–98.7)*	**14.1%** (10.9–17.7)*	60	31	367	1635
CRISPE‐14	14‐day death	6	6% (4.8–7.4)	**0.72** (0.59–0.86) *	**98.0%** (97.2–98.6)*	**14.7%** (11.3–18.7)*	56	35	324	1678
CRISPE‐14	14‐day death	6.5	6.5% (5.2–8.0)	**0.71** (0.58–0.85) *	**97.9%** (97.1–98.5)*	**15.6%** (11.9–19.8)*	54	37	293	1709
CRISPE‐14	14‐day death	7	7% (5.6–8.6)	**0.72** (0.58–0.85) *	**97.9%** (97.1–98.5)*	**16.4%** (12.5–20.9)*	53	38	270	1732
MELD 3.0	14‐day death	7	1.9% (1.4–2.6)	**0.32** (0.21–0.42) *	**97.9%** (94.6–99.4)*	**4.6%** (3.7–5.6)*	87	4	1818	184
MELD 3.0	14‐day death	8	2.0% (1.5–2.8)	**0.42** (0.31–0.53) *	**98.5%** (96.9–99.4)*	**5.2%** (4.1–6.3)*	84	7	1545	457
MELD 3.0	14‐day death	9	2.2% (1.6–2.9)	**0.46** (0.35–0.57) *	**98.5%** (97.1–99.3)*	**5.4%** (4.3–6.7)*	82	9	1427	575
MELD 3.0	14‐day death	10	2.3% (1.7–3.1)	**0.49** (0.37–0.60) *	**98.4%** (97.2–99.2)*	**5.7%** (4.6–7.1)*	80	11	1321	681
MELD 3.0	14‐day death	11	2.5% (1.9–3.3)	**0.52** (0.41–0.64) *	**98.4%** (97.3–99.1)*	**6.1%** (4.8–7.5)*	78	13	1204	798
MELD 3.0	14‐day death	12	2.7% (2.0–3.5)	**0.54** (0.42–0.66) *	**98.3%** (97.2–99.0)*	**6.4%** (5.0–7.9)*	75	16	1103	899
MELD 3.0	14‐day death	13	2.8% (2.2–3.7)	**0.57** (0.45–0.69) *	**98.2%** (97.2–98.9)*	**6.8%** (5.4–8.5)*	73	18	1002	1000
MELD 3.0	14‐day death	14	3.0% (2.4–3.9)	**0.58** (0.46–0.71) *	**98.2%** (97.2–98.9)*	**7.1%** (5.6–8.9)*	71	20	931	1071
MELD 3.0	14‐day death	15	3.3% (2.5–4.2)	**0.60** (0.47–0.73) *	**98.1%** (97.1–98.8)*	**7.6%** (5.9–9.5)*	68	23	831	1171
MELD 3.0	14‐day death	16	3.5% (2.7–4.4)	**0.59** (0.47–0.72) *	**97.9%** (96.9–98.6)*	**7.8%** (6.1–9.9)*	64	27	754	1248
MELD 3.0	14‐day death	17	3.7% (3.0–4.7)	**0.63** (0.50–0.75) *	**98.0%** (97.1–98.7)*	**8.5%** (6.6–10.7)*	64	27	689	1313
MELD 3.0	14‐day death	18	4.0% (3.2–5.0)	**0.61** (0.48–0.74) *	**97.8%** (96.9–98.5)*	**8.7%** (6.7–11.0)*	60	31	631	1371
MELD 3.0	14‐day death	19	4.3% (3.4–5.3)	**0.59** (0.45–0.72) *	**97.5%** (96.6–98.3)*	**8.8%** (6.7–11.3)*	55	36	571	1431
MELD 3.0	14‐day death	20	4.6% (3.7–5.6)	**0.57** (0.43–0.70) *	**97.4%** (96.4–98.1)*	**8.9%** (6.7–11.5)*	51	40	524	1478
MELD 3.0	14‐day death	21	4.9% (4.0–6.0)	**0.58** (0.44–0.72) *	**97.4%** (96.5–98.1)*	**9.8%** (7.3–12.7)*	49	42	452	1550
MELD 3.0	14‐day death	22	5.2% (4.2–6.4)	**0.59** (0.45–0.73) *	**97.4%** (96.5–98.1)*	**10.4%** (7.7–13.5)*	48	43	415	1587
MELD 3.0	14‐day death	23	5.6% (4.5–6.8)	**0.56** (0.42–0.70) *	**97.2%** (96.3–97.9)*	**10.8%** (7.9–14.2)*	43	48	357	1645
MELD 3.0	14‐day death	24	5.9% (4.8–7.3)	**0.58** (0.44–0.72) *	**97.2%** (96.3–97.9)*	**11.9%** (8.7–15.7)*	43	48	319	1683
MELD 3.0	14‐day death	25	6.3% (5.2–7.8)	**0.58** (0.44–0.71) *	**97.2%** (96.3–97.9)*	**12.6%** (9.2–16.7)*	41	50	284	1718
MELD 3.0	14‐day death	26	6.8% (5.5–8.3)	**0.58** (0.44–0.72) *	**97.2%** (96.3–97.9)*	**13.6%** (9.9–18.1)*	40	51	254	1748
ED disposition	30‐day death	Discharge	3.1% (2.1–4.4)	—	87.9% (85.9–89.7)	3.1% (2.0–4.4)	27	147	854	1065
CRISPE‐30	30‐day death	0.5	0.5% (0.3–0.8)	**0.33** (0.27–0.39) *	**100%** (94.9–100)*	**8.6% (7.4–9.9)** *	174	0	1849	70
CRISPE‐30	30‐day death	1	1% (0.7–1.5)	**0.44** (0.38–0.50) *	**100%** (98.7–100)*	**9.7% (8.3–11.1)** *	174	0	1628	291
CRISPE‐30	30‐day death	1.5	1.5% (1.0–2.1)	**0.51** (0.45–0.58) *	**99.4%** (98.1–99.9)*	**10.5% (9.1–12.1)** *	171	3	1455	464
CRISPE‐30	30‐day death	2	2% (1.5–2.7)	**0.57** (0.50–0.65) *	**98.7%** (97.5–99.5)*	**11.4% (9.8–13.2)** *	166	8	1288	631
CRISPE‐30	30‐day death	2.5	2.5% (1.9–3.3)	**0.62** (0.54–0.70) *	**98.3%** (97.2–99.1)*	**12.3% (10.6–14.2)** *	161	13	1149	770
CRISPE‐30	30‐day death	3	3% (2.3–3.9)	**0.67** (0.59–0.75) *	**98.4%** (97.4–99.1)*	**13.4% (11.5–15.5)** *	160	14	1032	887
CRISPE‐30	30‐day death	3.5	3.5% (2.7–4.5)	**0.69** (0.61–0.78) *	**98.2%** (97.1–98.9)*	**14.2% (12.2–16.4)** *	156	18	946	973
CRISPE‐30	30‐day death	4	4% (3.1–5.1)	**0.70** (0.61–0.78) *	**97.8%** (96.8–98.6)*	**14.6% (12.5–16.9)** *	151	23	882	1037
CRISPE‐30	30‐day death	4.5	4.5% (3.6–5.6)	**0.73** (0.64–0.82) *	**97.7%** (96.7–98.5)*	**15.8% (13.5–18.3)** *	148	26	790	1129
CRISPE‐30	30‐day death	5	5% (4.0–6.2)	**0.75** (0.66–0.84) *	**97.7%** (96.7–98.5)*	**16.6% (14.2–19.3)** *	146	28	731	1188
CRISPE‐30	30‐day death	5.5	5.5% (4.5–6.7)	**0.77** (0.68–0.86) *	**97.7%** (96.7–98.5)*	**17.7% (15.1–20.5)** *	145	29	674	1245
CRISPE‐30	30‐day death	6	6% (4.9–7.3)	**0.77** (0.67–0.86) *	**97.4%** (96.4–98.2)*	**18.2% (15.5–21.1)** *	140	34	631	1288
CRISPE‐30	30‐day death	6.5	6.5% (5.3–7.8)	**0.76** (0.67–0.86) *	**97.2%** (96.1–98.0)*	**18.8% (16.0–21.8)** *	135	39	584	1335
CRISPE‐30	30‐day death	7	7% (5.8–8.4)	**0.77** (0.68–0.86) *	**97.1%** (96.1–97.9)*	**19.6% (16.7–22.8)** *	133	41	545	1374
MELD 3.0	30‐day death	7	3.7% (2.9–4.7)	**0.33** (0.26–0.41) *	**95.2** (91.1–97.8)*	**8.7%** (7.4–10.0)*	165	9	1740	179
MELD 3.0	30‐day death	8	3.9% (3.1–5.0)	**0.43** (0.34–0.51) *	**96.3** (94.2–97.9)*	**9.6%** (8.2–11.2)*	157	17	1472	447
MELD 3.0	30‐day death	9	4.2% (3.4–5.3)	**0.46** (0.38–0.55) *	**96.4** (94.6–97.8)*	**10.1%** (8.7–11.8)*	153	21	1356	563
MELD 3.0	30‐day death	10	4.5% (3.7–5.6)	**0.49** (0.40–0.57) *	**96.2** (94.5–97.5)*	**10.6% (**9.0–12.3)*	148	26	1253	666
MELD 3.0	30‐day death	11	4.9% (4.0–6.0)	**0.54** (0.45–0.62) *	**96.5** (95.0–97.7)*	**11.4%** (9.7–13.3)*	146	28	1136	783
MELD 3.0	30‐day death	12	5.2% (4.3–6.4)	**0.56** (0.47–0.65) *	**96.4** (95.0–97.5)*	**12.0%** (10.2–14.0)*	141	33	1037	882
MELD 3.0	30‐day death	13	5.6% (4.6–6.8)	**0.59** (0.50–0.68) *	**96.4 (**95.0–97.4)*	**12.7%** (10.8–14.9)*	137	37	938	981
MELD 3.0	30‐day death	14	6.0% (5.0–7.2)	**0.60** (0.50–0.69) *	**96.2** (94.8–97.2)*	**13.2%** (11.1–15.4)*	132	42	870	1049
MELD 3.0	30‐day death	15	6.4% (5.4–7.6)	**0.60** (0.51–0.70) *	**95.9** (94.6–96.9)*	**13.9%** (11.7–16.3)*	125	49	774	1145
MELD 3.0	30‐day death	16	6.9% (5.8–8.1)	**0.61** (0.51–0.71) *	**95.7 (**94.4–96.7)*	**14.5% (**12.2–17.2)*	119	55	699	1220

*Note*: All test characteristics reported as estimate (95% CI) unless otherwise indicated. Bold: statistically significant at *p* < 0.05.

Abbreviations: CRISPE‐14, Cirrhosis Risk Instrument for Stratifying Post‐Emergency department mortality 14‐day mortality model; CRISPE‐30, Cirrhosis Risk Instrument for Stratifying Post‐Emergency department mortality 30‐day mortality model; FN, false negative; FP, false positive; MELD, Model for End‐Stage Liver Disease; NRI, net reclassification index; NPV, negative predictive value; PPV, positive predictive value; TP, true positive; TN, true negative.

^a^ Score range is 0–100 for CRISPE‐14 and CRISPE‐30, and equal to predicted probability of the outcome. MELD 3.0 is an integer score with minimum of 6 (i.e., <7 is the lowest possible cutoff).

^b^ Compared to ED disposition.

^c^ Blue = net reclassification improvement; red = worse after reclassification (compared to classification by ED disposition).

* *p* < 0.05 compared to ED disposition.

At every cutoff of CRISPE‐14 (0.5–7.0), CRISPE‐30 (0.5–7.0), and MELD 3.0 (7–26) with a ≤7% predicted probability of 30‐day mortality, the risk scores achieved a significant improvement in NPV and PPV compared to ED disposition (Table 4). NPV of ≥99% was observed with any CRISPE‐14 score below 2.5 (56.3% of cohort). The highest NPV for MELD 3.0 and 14‐day death was 98.5%, observed at MELD 3.0 <9 (27.9% of cohort; Table 4 and Figure 2).

CRISPE‐14, CRISPE‐30, and MELD 3.0 achieved significant (*p* < 0.05) NRI compared to ED disposition at each observed cutoff (Table 4), both overall and for decedents specifically. The CDIs yielded net appropriate reclassification of survivors, decedents, and overall ED disposition compared to usual care (Table 4). At a cutoff of <2.5 (Table 4 and Figure 2B), CRISPE‐14 was associated with net appropriate reclassification in +4.6% of ED dispositions (NND 22). CRISPE‐30 at the <4.5 cutoff was associated with improvement in +8.8% of ED dispositions (NND 12; Table 4 and Figure 2A). CRISPE‐14 and CRISPE‐30 at these cutoffs would have yielded improvement of ED disposition for 71.4% and 69.5% deaths (14‐day and 30‐day, respectively; NND 2). At MELD 3.0 <15, the NNDs were 28 and 84 patients, for 14‐ and 30‐day death, respectively.

## DISCUSSION

Individuals with cirrhosis present a significant challenge for risk stratification and disposition decision making by ED physicians and the hepatologists they may consult.^3^, ^6^, ^7^ Our study, utilizing a cohort of over 2000 individuals across 16 sites within a statewide health care system, has three key findings which overall suggest that CDIs may help clinicians with these difficult risk‐stratification decisions. First, we developed and internally validated the novel CRISPE CDI specifically for use in the ED patients, demonstrating several readily available ED clinical variables are strongly predictive of mortality in cirrhosis patients. Second, we externally validated the MELD 3.0 score for predicting all‐cause mortality in ED patients. Third, and most critically, both CRISPE and MELD 3.0 outperformed ED disposition under usual care decision making. In total, our results support the utility of CDIs for aiding physicians’ decision making in risk stratifying undifferentiated emergency patients with cirrhosis, for the overall goal of avoiding adverse events while optimizing HCU.

The CRISPE tool demonstrated robust performance for predicting short‐term mortality using ED variables, with AUROCs exceeding 0.800 across sensitivity analyses and conditions (Figure 2). CRISPE‐14 and CRISPE‐30 exhibited exceptional NPV for 14‐ and 30‐day mortality across cutoffs, while improving PPV compared to ED disposition (Table 4). CRISPE‐14 and CRISPE‐30 demonstrated favorable net reclassification of actual ED dispositions (Table 4), suggesting the potential for real‐world impact if CRISPE is externally validated. This was especially true for patients who died within 14 or 30 days, in whom application of CRISPE would have yielded net reclassification improvement of disposition (i.e., from discharge to admission) in just one out of every two decedents. CRISPE has high flexibility for varying conditions of ED use given that we trained and tested the model using individuals regardless of reason for the ED presentation, making the tool useful regardless of ED diagnostic accuracy for challenging to recognize and/or heterogenous specific acute syndromes (e.g., altered mental status, sepsis, GI bleeding). The CRISPE‐14‐EMR and CRISPE‐30‐EMR versions, which removed all potentially subjective variables, performed similarly strong and suggest potential for direct EMR integration. The observation that removing all but the most objective variables (age, vital signs, lab studies) retained the tools’ overall accuracy may also reassure usefulness for clinicians who are skeptical of CDIs that incorporate greater subjectivity. The robust performance of the CRISPE variants is highly promising, especially given superior performance compared to both ED disposition and MELD 3.0, but external validation is required prior to clinical use. The development and internal validation of CRISPE highlights specific variables readily available to clinicians, which strongly predict post‐ED mortality for patients with cirrhosis. Many of these variables are not captured in the existing MELD score variants. In particular, lack of established outpatient hepatology/GI care, abnormal vital signs, comorbid cancer, and altered mental status were all significant multivariable adjusted predictors of 30‐day mortality for all‐cause visits in cirrhosis patients. Consideration of these factors as correlates of short‐term mortality, alongside traditional laboratory markers like albumin and bilirubin, is warranted in evaluating cirrhosis patients in the ED.

We also externally validated the performance of MELD 3.0 and previous variants in the ED population for short‐term post‐ED mortality prediction. MELD 3.0 demonstrated moderate discrimination by AUROC for both 14‐ and 30‐day mortality and outperformed ED disposition (Figure 2). This is not surprising given that prior studies have shown excellent performance of MELD and its iterations in predicting mortality in non‐ED settings such as liver transplant list prioritization, post‐TIPS placement, and perioperative risk‐stratification.^15^, ^16^, ^26^ Our study is the first to validate its use in ED patients. The performance of MELD 3.0 at its most sensitive cutoffs (MELD 3.0 15, 9, 7) may or may not meet the threshold for “rule out” that ED physicians are generally comfortable with (Figure 2 and Table 4). In other conditions for which CDIs are used to risk stratify ED patients, “low risk” generally has been used to refer to event rates of around 2%–3%.^8^, ^9^, ^10^, ^11^, ^23^, ^24^ For instance, “low” versus “moderate” risk in the CURB‐65 score for pneumonia are 0.6%–2.7% versus 2.7%–6.8%, respectively, for 30‐day mortality.^10^ In heart failure, a chronic condition that like cirrhosis can have deadly acute exacerbations and accounts for a large number of acute care visits to the ED, ED physicians have cited an “acceptable miss rate” of ≤3.8% mortality at 30 days as the threshold below which they would feel comfortable discharging a patient.^27^ MELD 3.0 predicted 3.7% 30‐day mortality risk and 1.9% 14‐day mortality risk at the most sensitive cutoff of 7 (i.e., given a minimum score of 6). Notably, this accounted for only 9% of the cohort; by contrast CRISPE's most sensitive cutoffs (CRISPE‐30 1.0 and CRISPE‐14 0.5, ≤ 1% mortality) accounted for nearly 50% of the cohort (Figure 2 and Table 4). Nevertheless, MELD 3.0 outperformed ED disposition at this and other cutoffs just like CRISPE (Table 2). With this in mind, physicians may find MELD 3.0 helpful for aiding risk stratification and disposition decision making, and unlike CRISPE the MELD 3.0 score has now been externally validated for ED use.

It is critical to note that MELD 3.0 did not perform well when labs were not obtained by the ED physician (see sensitivity analyses in the supplement). CRISPE, by contrast, had similarly robust performance under the assumption of standardized normal values when a lab was not obtained. This is not surprising, since five of the seven variables in MELD 3.0 are laboratory variables, compared to lesser influence of lab values in CRISPE. Nevertheless, it does underscore the prognostic importance of the MELD 3.0 labs in patients seeking ED care (serum Na, creatinine, bilirubin, albumin, and INR) regardless of reason for presentation. Moreover, it shows that MELD 3.0 should not be used clinically without obtaining each lab (e.g., under assumptions of “normality”).

CRISPE incorporates several variables related to acuity of emergent presentations not captured by MELD but well suited to the ED, such as age, vital signs, symptoms of critical acute syndromes such as volume overload or sepsis, and comorbidities common in cirrhosis that increase the risk of acute care problems (e.g., cancer).^28^, ^29^, ^30^, ^31^, ^32^ The tool also emphasized patient characteristics that likely affect care navigation after an ED visit such as within‐system GI/hepatology follow‐up. Prior studies have demonstrated the benefit of specialty follow‐up^33^, ^34^ but literature evaluating follow‐up within or outside the same system as the ED is limited. Our study is unique in examining this aspect of specialty care coordination. HCC and other comorbid cancers, seen at higher rates than the general population,^35^ also emerged as significant predictors of short‐term mortality in CRISPE‐30. Traditional models like the MELD variants do not incorporate HCC and may underestimate mortality, since patients with HCC often maintain lower MELD‐Na scores until very advanced stages.^36^ Interestingly, active alcohol use at ED presentation was a protective factor, and while at first glance this may seem paradoxical, we suspect that this reflects a reality that patients with cirrhosis who are healthy enough to continue drinking alcohol are likely more well overall. In particular, we suspect that this relationship reflects patients whose reason for presentation to the ED was alcohol intoxication and/or acute‐on‐chronic abdominal pain exacerbated by alcohol, as opposed to more serious reasons for ED use such as sepsis or hepatic encephalopathy. Our data emphasize the importance of a comprehensive tool such as CRISPE incorporating diverse sources of risk.

## LIMITATIONS

Our study has several limitations. First, the retrospective nature carries limitations. While we attempted to mitigate some of these limitations (e.g., manual chart review by hepatology experts blinded to the analysis, sensitivity analyses to evaluate missing data assumptions, etc.; see Data S1), a prospective study is warranted. In particular, inherently subjective variables like the reason for ED visit and social drivers were primarily derived from clinical documentation, and such data elements could be less accurate than what could be obtained in a prospective study. As with any subjective characteristic in a CDI, this could introduce uncertainty. The small difference in performance between the more objective CRISPE‐EMR and the full CRISPE scores helps to reassure that any such bias is relatively small. Nevertheless, EMR integration itself may be a challenge due to implementation issues such as variable attribution, clinician adherence, optimization for clinical workflows, and training needed to understand and use the tool appropriately. An implementation trial is needed to evaluate real‐world use of the EMR‐specific and full‐scale CRISPE tools. Second, we assumed a normal value for laboratory studies if they were not ordered, based on a similar approach being applied in the development of prior robust CDIs in the ED setting.^23^, ^24^ This was done to allow flexibility of use in a future external validation of CRISPE (i.e., allowing use of the tool without mandating all lab components be obtained every time), and sensitivity analysis showed similar performance with versus without this assumption (supplement). Third, data were extracted during the period of 2021, which could suggest a potential impact of COVID‐19 on ED visits. However, only 3% of the individuals with ED visit had COVID‐19 pneumonia. This was described in detail in our comprehensive paper on ED utilization of individuals with cirrhosis.^19^ Fourth, despite robust techniques for bias penalization and internal validation throughout the development process for CRISPE, an external validation study is needed prior to utilization in clinical practice. Until then, clinicians may find MELD 3.0 useful, as it is an existing CDI that we have now validated in the ED setting for ED‐relevant outcomes. As with any CDI, disposition decisions are multifactorial and not solely driven by risk of post‐ED mortality. One aspect we did not analyze is whether reclassification performance for CRISPE and MELD varied between clinical sites (e.g., better reclassification at one site where baseline classification was poor, compared to another where usual care already achieved strong classification). Another parallel and inherent limitation of mortality CDIs is that risk of mortality does not always have a one‐to‐one relationship with optimal ED disposition. Examples would include patients with a high but nonmodifiable (i.e., with hospitalization vs. outpatient care) risk of death due to severe chronic disease and/or patients for whom the optimal clinical trajectory is palliative or hospice care. With these points in mind, MELD 3.0 and CRISPE should be considered as adjuncts to clinical judgment and not replacements for it. Finally, although the study cohort was representative of a statewide population including urban and rural settings, it lacked racial and ethnic diversity, which therefore warrants further study in demographic minority subgroups.

## CONCLUSIONS

Clinical decision instruments may be useful for aiding risk stratification of all‐cause ED encounters among patients with cirrhosis for 14‐ and 30‐day mortality. Both the Cirrhosis Risk Instrument for Stratifying Post‐Emergency department mortality (CRISPE) and Model for End‐Stage Liver Disease (MELD) 3.0 favorably net reclassified ED disposition, were particularly strong at reclassifying decedents, and improved negative and positive predictive value compared to disposition decisions. While the former needs external validation prior to clinical use, MELD 3.0 is now externally validated in an ED setting. Through improved risk stratification, clinical decision instruments may aid clinicians in differentiating high‐risk patients needing hospitalizations and/or higher levels of care (e.g., intensive care unit) from those low‐risk patients who can potentially be discharged after ED management alone.

## AUTHOR CONTRIBUTIONS

Study concept and design: Swetha Parvataneni, Archita P. Desai, Nicholas Eric Harrison. Data retrieval: Swetha Parvataneni, Yara Sarkis, Michelle Haugh, Brittany Baker, Archita P. Desai. Data analysis: Swetha Parvataneni, Archita P. Desai, Nicholas Eric Harrison. Manuscript preparation: Swetha Parvataneni, Archita P. Desai, Nicholas Eric Harrison. Critical manuscript review: all authors.

## FUNDING INFORMATION

Dr. Swetha Parvataneni is in part supported by institutional funding through David W. Crabb Professorship and Terence Kahn Liver Research Program. Dr. Desai is supported by K23 DK123408. Dr. Harrison is supported by KL2TR002530 (Sheri L. Robb, PI) and UL1TR002529 (Sharon M. Moe and Sarah E. Wiehe, co‐PIs) from the National Institutes of Health, National Center for Advancing Translational Sciences, Clinical and Translational Sciences Award.

## CONFLICT OF INTEREST STATEMENT

The authors have no conflicts of interest to report. For full disclosure, Dr. Chalasani reports consulting agreements with Madrigal, Zydus, GSK, Ipsen, Merck, Pfizer, Altimmune, and Ventyx. He received research grant support from DSM and Exact Sciences. He serves on the board of Avant Sante, LLC and has equity interest in that contract research organization. Dr. Harrison reports consulting and honoraria from Vave Health and EB Medicine. He has received research grant support from Abbott, Siemens, NIH NCATS, the Doris Duke Foundation, Blue Cross Blue Shield of Michigan Foundation, and the Indiana CTSI. The remaining authors have nothing to disclose.

## Supporting information


Data S1.

## Data Availability

The analytic methods used in this study are detailed in the methods. Due to HIPAA regulations, individual level data are not available publicly from this retrospective study. However, interested investigators are encouraged to contact the corresponding author for appropriate deidentified data sharing.
