# Supplementary material for: Clinical decision instruments for predicting mortality in patients with cirrhosis seeking emergency department care
Source: Acad Emerg Med. 2025 Jan 8;32(6):604–18. doi: 10.1111/acem.15088 (PMC12171671; doi:10.1111/acem.15088)
Supplement: Supplementary file 1 — Data S1. [file ACEM-32-604-s001.docx]

**Supplementary appendix:**

**Contents**

1.Methods

a. Data variables

b. Statistical analysis

2. Tables

Supplementary Table 1: ICD 10 codes of cirrhosis and comorbidities in Elixhauser comorbidity index and predictors included in LASSO.

Supplementary Table 2: Transparent Reporting of a multivariable prediction model for individual prognosis or diagnosis development and validation checklist for CRISPE.

Supplementary Table 3: Transparent Reporting of a multivariable prediction model for individual prognosis or diagnosis validation checklist for MELD.

3. References

**Supplemental Methods:**

**Data variables:**

A dataset of 119 variables was assembled for included patients. Baseline demographic information, HCU in the 12 months before the ED visit (number of ED encounters, inpatient encounters, outpatient encounters, no-show visits), labs obtained during the ED visit were extracted from the data warehouse. ED-level variables within 8 hours of arrival (vital signs, laboratory studies, blood cultures, urine studies, imaging, procedures, ED consultations), and ED disposition (discharge to home, observation, hospitalization, against medical advice, death, transfer), were retrieved from the data warehouse and confirmed through chart review. The Elixhauser comorbidity index (ECI) was calculated using ICD-10 codes (Supplementary Table 1), while geocoded home-addresses were used to determine the social deprivation index (SDI) (neighborhood-level social determinant of health) (1, 2).

Manual chart review by clinical study investigators blinded to the analysis was conducted to extract cirrhosis-related characteristics and complications, social determinants prompting the ED visit, reasons for the initial ED visit, discharge diagnosis for hospitalized patients, and follow-up appointments post-discharge.

**Handling of missingness:**

Frequency of missing lab values and AUROC for models accounting for missing individual missing values or any missingness are presented below.

|  | | 14-day Mortality | | 30-day Mortality | |
| --- | --- | --- | --- | --- | --- |
| Lab missing (not obtained by physician) | % Missing | CRISPE-14 | MELD 3.0 | CRISPE-30 AUC | MELD 3.0 |
| Any missing | 55% | 0.836 | 0.692 | 0.806 | 0.646 |
| Na | 14% | 0.764 | 0.597 | 0.797 | 0.585 |
| Creatinine | 14% | 0.764 | 0.596 | 0.799 | 0.595 |
| Bili | 24% | 0.785 | 0.527 | 0.755 | 0.529 |
| Albumin | 24% | 0.782 | 0.527 | 0.758 | 0.529 |
| INR | 51% | 0.846 | 0.691 | 0.818 | 0.650 |

Given the shared EMR, reason for missingness was due to the lab not being ordered by the treatment team in all cases. To account for these missing labs, we used standardized normal values within the reference range (creatinine 0.9 mEq/L, sodium 140 mEq/L, INR 1.0, albumin 4.0 g/dL, total bilirubin 0.5 mg/dL). This approach was chosen to align with practices in the development of prior ED risk-stratification tools, to allow clinical flexibility (i.e. allow use when a certain lab was not obtained) and avoid biasing the model by missingness not at random (MNAR). First, using lab values obtained before ED arrival or after ED disposition (including during hospitalization) would reduce validity for use of the CDI specifically in ED patients.  Second, we suspected that missing data procedures like multiple imputation would not be valid here since the decision to order or not order a given test likely has strong correlation with reason for ED presentation, ED providers’ usual care decision making, illness severity of the patient, and ultimately the outcomes of interest (i.e. mortality). As such, laboratory variables are potentially Not Missing at Random (NMAR) and therefore not appropriate for multiple imputation. Third, use of a default “normal” value in the case of a lab not being obtained is consistent with derivation practices in other ED-based CDIs (e.g. use of troponin in the Canadian Syncope Risk Score) which subsequently validated with strong performance (3, 4). This approach has certain intuitive and pragmatic benefits for CDIs in the ED. ED physician decisions to not order a lab associated with adverse events are more likely in patients who are younger, have less comorbidities, and lower adverse event rates (3). Thus, the assumption of a standard normal value when a lab is not ordered aligns with both ED provider gestalt and true adverse event risk, specifically in the most obviously healthy and lowest risk patients. Moreover, this also means that any inaccuracy of the CDI by assuming a normal value is concentrated in the training and validation data within the patient subgroup for whom clinicians are least likely to need a CDI (because usual care judgement is sufficient to identify low-risk) and away from those in whom a CDI would most benefit the clinician (i.e. uncertainty of usual care gestalt risk-stratification, represented by the clinician ordering a broader workup). Scores for the 3 variations of MELD were calculated under the same assumptions for missing laboratory variables as CRISPE to facilitate a like to like comparison.

**Supplementary Table 1:**

| Variable | ICD-10 code^#^ |
| --- | --- |
| **Cirrhosis** | K74.3, K74.4, K74.5, K74.6x, K70.3x, K71.7 |
| **Liver transplant*** | 47135,47143,47144 |
| **Elixhauser Comoribidity Index** | ICD 10 CODE |
| **Obesity** | E66.0 |
| **Peripheral vascular disease** | I73.1, I73.8, I73.9, I77.1, 'I79.0', 'I79.2', 'K55.1', 'K55.8', 'K55.9', 'Z95.8', 'Z95.9' |
| **Blood loss anemia** | D50.0 |
| **Psychoses** | F30.2, 'F31.2', 'F31.5 |
| **Drug abuse** | Z71.5', 'Z72.2 |
| **Alcohol abuse** | 'G62.1', 'I42.6', 'K29.2', 'K70.0', 'K70.3', 'K70.9', 'Z50.2', 'Z71.4', 'Z72.1 |
| **Deficiency anemia** | D50.8', 'D50.9 |
| **Fluid electrolyte disorders** | E22.2 |
| **Weight loss** | R63.4', 'R64 |
| **Coagulopathy** | D69.1', 'D69.3', 'D69.6 |
| **Rheumatoid arthritis** | L94.0', 'L94.1', 'L94.3', 'M12.0', 'M12.3', 'M31.0', 'M31.1', 'M31.2', 'M31.3', 'M46.1', 'M46.8', 'M46.9 |
| **Renal failure** | I12.0', 'I13.1', 'Z94.0', 'Z99.2', 'N25.0', 'Z49.0', 'Z49.1', 'Z49.2 |
| **Metastatic cancer** | C77, C78, C79, C80 |
| **Lymphoma** | C90.0, C90.2 |
| **AIDS/HIV** | B20, B21, B22, B24 |
| **Peptic ulcer disease** | K25.7', 'K25.9', 'K26.7', 'K26.9', 'K27.7', 'K27.9', 'K28.7', 'K28.9 |
| **Congestive Heart failure** | 'I09.9', 'I11.0', 'I13.0', 'I13.2', 'I25.5', 'I42.0', 'I42.5', 'I42.6', 'I42.7', 'I42.8', 'I42.9', 'P29.0 |
| **Cardiac Arrhythmia** | I44.1', 'I44.2', 'I44.3', 'I45.6', 'I45.9', 'R00.0', 'R00.1', 'R00.8', 'T82.1', 'Z45.0', 'Z95.0 |
| **Valvular disease** | A52.0', 'I09.1', 'I09.8', 'Q23.0', 'Q23.1', 'Q23.2', 'Q23.3', 'Z95.2', 'Z95.3', 'Z95.4 |
| **Pulmonary circulation disorders** | I28.0', 'I28.8', 'I28.9 |
| **Hypertension uncomplicated** | I10 |
| **Hypertension complicated** | I11, I12, I13, I15 |
| **Paralysis** | G04.1', 'G11.4', 'G80.1', 'G80.2', 'G83.0', 'G83.1', 'G83.2', 'G83.3', 'G83.4', 'G83.9 |
| **Other neurological disorders** | G25.4', 'G25.5', 'G31.2', 'G31.8', 'G31.9', 'G93.1', 'G93.4', 'R47.0 |
| **Chronic pulmonary disease** | I27.8', 'I27.9', 'J68.4', 'J70.1', 'J70.3 |
| **Diabetes, uncomplicated** | E10.0', 'E10.1', 'E10.9', 'E11.0', 'E11.1', 'E11.9', 'E12.0', 'E12.1', 'E12.9', 'E13.0', 'E13.1', 'E13.9', 'E14.0', 'E14.1', 'E14.9 |
| **Diabetes, complicated** | E10.2', 'E10.3', 'E10.4', 'E10.5', 'E10.6', 'E10.7', 'E10.8', 'E11.2', 'E11.3', 'E11.4', 'E11.5', 'E11.6', 'E11.7', 'E11.8', 'E12.2', 'E12.3', 'E12.4', 'E12.5', 'E12.6', 'E12.7', 'E12.8', 'E13.2', 'E13.3', 'E13.4', 'E13.5', 'E13.6', 'E13.7', 'E13.8', 'E14.2', 'E14.3', 'E14.4', 'E14.5', 'E14.6', 'E14.7', 'E14.8 |
| **Hypothyroid** | E89.0 |
| **Predictors included in the LASSO** |  |
| Baseline demographics | Age, male, history of liver transplant, diagnosis of cirrhosis (1. Clinical examination noted in history by GI or hepatology provider, 2. Imaging showing nodular liver with ascites or varices/collaterals or Hepatocellular carcinoma or splenomegaly with low platelets, 3. Liver Biopsy), new diagnosis of cirrhosis during the ED visit, Living (Home), Social divers for ED visit (none, active alcohol use, active substance use, non-adherence to medications/diet at home, transport issues, caregiver issues, unable to get visit with primary care physician, unable to ger visit with specialist in time, other) |
| GI Follow up | Follow up with GI within the Indiana University health (IUH), follow up with GI outside the IUH, Unknown, any known GI. |
| Baseline cirrhosis characteristics | Ascites- no ascites, controlled (if on diuretics, history of TIPS and no large volume paracentesis in previous 3 months), Uncontrolled.  Hepatic encephalopathy (HE)- no HE, controlled on lactulose and rifaximin, uncontrolled.  Varices- esophageal, gastric. Known variceal bleeding, known TIPS, known HCC, known dialysis. |
| Comorbidities of the Elixhauser Comorbidity Index | Alcohol abuse, AIDS/HIV, Cardiac arrythmia, Blood loss anemia, Congestive heart failure. Chronic pulmonary disease, coagulopathy, anemia deficiency, depression, diabetes with chronic complications, diabetes without chronic complications, drug abuse, fluid and electrolyte disorders, Hypertension complicated, hypertension uncomplicated, hypothyroidism, other neurological disorders, obesity, paralysis, pulmonary circulation disorder, rheumatoid arthritis, renal failure, solid tumor without metastasis, valvular disease, weight loss |
| Reasons for ED visit | Ascites/abdominal distension, concern for spontaneous bacterial peritonitis, lower extremity edema/ volume overload, shortness of breath, altered mental status, fever, abdominal pain, chest pain, nausea/vomiting, abnormal labs, abnormal imaging, GI bleeding (melena, hematemesis, hematochezia), doctor instructions, upper respiratory symptoms, diarrhea, other GI symptoms, stroke like symptoms/ seizures, urinary symptoms, trauma/injury/fracture, musculoskeletal pain/swelling, fall/generalized weakness, presyncope/syncope, cardiac arrest/respiratory arrest, abnormal vitals, non-alcohol drug intoxication/withdrawal, alcohol related abuse, alcohol withdrawal, alcohol detox, psychiatric, other. |
| Prior healthcare utilization (12 months prior to the ED visit) | Prior emergency encounters, prior inpatient encounters, prior no shows, prior outpatient encounters. |
| Labs during the initial ED visit | Sodium, bilirubin, creatinine, INR, albumin |
| Vitals during the ED visit | Heart rate, respiratory rate, temperature, oxygen saturation, systolic blood pressures, diastolic blood pressure, shock index, pulse pressure |
| Other | Business hours, long weekend, longer weekend, weekend |

**Supplementary Table 2: Transparent Reporting of a multivariable prediction model for individual prognosis or diagnosis Development and Validation Checklist for CRISPE.**

| **Section/Topic** | **Item** |  | **Checklist Item** | **Page** |
| --- | --- | --- | --- | --- |
| **Title and abstract** | | | | |
| Title | 1 | D;V | Identify the study as developing and/or validating a multivariable prediction model, the target population, and the outcome to be predicted. | 1 |
| Abstract | 2 | D;V | Provide a summary of objectives, study design, setting, participants, sample size, predictors, outcome, statistical analysis, results, and conclusions. | 4,5 |
| **Introduction** | | | | |
| Background and objectives | 3a | D;V | Explain the medical context (including whether diagnostic or prognostic) and rationale for developing or validating the multivariable prediction model, including references to existing models. | 6 |
|  | 3b | D;V | Specify the objectives, including whether the study describes the development or validation of the model or both. | 6,7 |
| **Methods** | | | | |
| Source of data | 4a | D;V | Describe the study design or source of data (e.g., randomized trial, cohort, or registry data), separately for the development and validation data sets, if applicable. | 7 |
|  | 4b | D;V | Specify the key study dates, including start of accrual; end of accrual; and, if applicable, end of follow-up. | 7 |
| Participants | 5a | D;V | Specify key elements of the study setting (e.g., primary care, secondary care, general population) including number and location of centers. | 7 |
|  | 5b | D;V | Describe eligibility criteria for participants. | 7 |
|  | 5c | D;V | Give details of treatments received, if relevant. | 7 |
| Outcome | 6a | D;V | Clearly define the outcome that is predicted by the prediction model, including how and when assessed. | 8 |
|  | 6b | D;V | Report any actions to blind assessment of the outcome to be predicted. | NA |
| Predictors | 7a | D;V | Clearly define all predictors used in developing or validating the multivariable prediction model, including how and when they were measured. | 9,10 |
|  | 7b | D;V | Report any actions to blind assessment of predictors for the outcome and other predictors. | 7,8 |
| Sample size | 8 | D;V | Explain how the study size was arrived at. | 7, 8, Figure 1 |
| Missing data | 9 | D;V | Describe how missing data were handled (e.g., complete-case analysis, single imputation, multiple imputation) with details of any imputation method. | 9, Supplement |
| Statistical analysis methods | 10a | D | Describe how predictors were handled in the analyses. | 9,10 |
|  | 10b | D | Specify type of model, all model-building procedures (including any predictor selection), and method for internal validation. | 10,11 |
|  | 10c | V | For validation, describe how the predictions were calculated. | 11 |
|  | 10d | D;V | Specify all measures used to assess model performance and, if relevant, to compare multiple models. | 11,12 |
|  | 10e | V | Describe any model updating (e.g., recalibration) arising from the validation, if done. | 10,11 |
| Risk groups | 11 | D;V | Provide details on how risk groups were created, if done. | 10,11 |
| Development vs. validation | 12 | V | For validation, identify any differences from the development data in setting, eligibility criteria, outcome, and predictors. | 10,11 |
| **Results** | | | | |
| Participants | 13a | D;V | Describe the flow of participants through the study, including the number of participants with and without the outcome and, if applicable, a summary of the follow-up time. A diagram may be helpful. | Figure 1 |
|  | 13b | D;V | Describe the characteristics of the participants (basic demographics, clinical features, available predictors), including the number of participants with missing data for predictors and outcome. | Table 1 |
|  | 13c | V | For validation, show a comparison with the development data of the distribution of important variables (demographics, predictors and outcome). | NA |
| Model development | 14a | D | Specify the number of participants and outcome events in each analysis. | 13 |
|  | 14b | D | If done, report the unadjusted association between each candidate predictor and outcome. | Table 3 |
| Model specification | 15a | D | Present the full prediction model to allow predictions for individuals (i.e., all regression coefficients, and model intercept or baseline survival at a given time point). | Table 3 |
|  | 15b | D | Explain how to the use the prediction model. | 13 |
| Model performance | 16 | D;V | Report performance measures (with CIs) for the prediction model. | Table 3 |
| Model-updating | 17 | V | If done, report the results from any model updating (i.e., model specification, model performance). | 13,14 |
| **Discussion** | | | | |
| Limitations | 18 | D;V | Discuss any limitations of the study (such as nonrepresentative sample, few events per predictor, missing data). | 19,20,21 |
| Interpretation | 19a | V | For validation, discuss the results with reference to performance in the development data, and any other validation data. | 16,17,18 |
|  | 19b | D;V | Give an overall interpretation of the results, considering objectives, limitations, results from similar studies, and other relevant evidence. | 16,17,18,19 |
| Implications | 20 | D;V | Discuss the potential clinical use of the model and implications for future research. | 21 |
| **Other information** | | | | |
| Supplementary information | 21 | D;V | Provide information about the availability of supplementary resources, such as study protocol, Web calculator, and data sets. | NA |
| Funding | 22 | D;V | Give the source of funding and the role of the funders for the present study. | 2 |

**Supplementary Table 3: Transparent Reporting of a multivariable prediction model for individual prognosis or diagnosis Validation Checklist for MELD.**

| **Section/Topic** | **Item** | **Checklist Item** | **Page** |
| --- | --- | --- | --- |
| **Title and abstract** | | | |
| Title | 1 | Identify the study as developing and/or validating a multivariable prediction model, the target population, and the outcome to be predicted. | 1 |
| Abstract | 2 | Provide a summary of objectives, study design, setting, participants, sample size, predictors, outcome, statistical analysis, results, and conclusions. | 4,5 |
| **Introduction** | | | |
| Background and objectives | 3a | Explain the medical context (including whether diagnostic or prognostic) and rationale for developing or validating the multivariable prediction model, including references to existing models. | 6 |
|  | 3b | Specify the objectives, including whether the study describes the development or validation of the model or both. | 6,7 |
| **Methods** | | | |
| Source of data | 4a | Describe the study design or source of data (e.g., randomized trial, cohort, or registry data), separately for the development and validation data sets, if applicable. | 7 |
|  | 4b | Specify the key study dates, including start of accrual; end of accrual; and, if applicable, end of follow-up. | 7 |
| Participants | 5a | Specify key elements of the study setting (e.g., primary care, secondary care, general population) including number and location of centers. | 7 |
|  | 5b | Describe eligibility criteria for participants. | 7,8 |
|  | 5c | Give details of treatments received, if relevant. | NA |
| Outcome | 6a | Clearly define the outcome that is predicted by the prediction model, including how and when assessed. | 8 |
|  | 6b | Report any actions to blind assessment of the outcome to be predicted. | 8 |
| Predictors | 7a | Clearly define all predictors used in developing or validating the multivariable prediction model, including how and when they were measured. | 9,10 Supplement table 1 |
|  | 7b | Report any actions to blind assessment of predictors for the outcome and other predictors. | 8 |
| Sample size | 8 | Explain how the study size was arrived at. | Figure 1 |
| Missing data | 9 | Describe how missing data were handled (e.g., complete-case analysis, single imputation, multiple imputation) with details of any imputation method. | 9,Supplement |
| Statistical analysis methods | 10c | For validation, describe how the predictions were calculated. | 10,11 |
|  | 10d | Specify all measures used to assess model performance and, if relevant, to compare multiple models. | 11,12 |
|  | 10e | Describe any model updating (e.g., recalibration) arising from the validation, if done. | 11,12 |
| Risk groups | 11 | Provide details on how risk groups were created, if done. | NA |
| Development vs. validation | 12 | For validation, identify any differences from the development data in setting, eligibility criteria, outcome, and predictors. | 10,11 |
| **Results** | | | |
| Participants | 13a | Describe the flow of participants through the study, including the number of participants with and without the outcome and, if applicable, a summary of the follow-up time. A diagram may be helpful. | Figure 1 |
|  | 13b | Describe the characteristics of the participants (basic demographics, clinical features, available predictors), including the number of participants with missing data for predictors and outcome. | Figure 1 Table 1 |
|  | 13c | For validation, show a comparison with the development data of the distribution of important variables (demographics, predictors and outcome). | NA |
| Model performance | 16 | Report performance measures (with CIs) for the prediction model. | 13,14,Figure 2, Table 4 |
| Model-updating | 17 | If done, report the results from any model updating (i.e., model specification, model performance). | NA |
| **Discussion** | | | |
| Limitations | 18 | Discuss any limitations of the study (such as nonrepresentative sample, few events per predictor, missing data). | 19,20,21 |
| Interpretation | 19a | For validation, discuss the results with reference to performance in the development data, and any other validation data. | 16,17,18 |
|  | 19b | Give an overall interpretation of the results, considering objectives, limitations, results from similar studies, and other relevant evidence. | 16,17,18,19 |
| Implications | 20 | Discuss the potential clinical use of the model and implications for future research. | 21 |
| **Other information** | | | |
| Supplementary information | 21 | Provide information about the availability of supplementary resources, such as study protocol, Web calculator, and data sets. | NA |
| Funding | 22 | Give the source of funding and the role of the funders for the present study. | 2 |

**REFERENCES:**

1. Giammarino AM, Qiu H, Bulsara K, Khan S, Jiang Y, Da BL, et al. Community Socioeconomic Deprivation Predicts Nonalcoholic Steatohepatitis. Hepatol Commun. 2022;6(3):550-60.

2. Butler DC, Petterson S, Phillips RL, Bazemore AW. Measures of social deprivation that predict health care access and need within a rational area of primary care service delivery. Health Serv Res. 2013;48(2 Pt 1):539-59.

3. Venkatesh Thiruganasambandamoorthy KK, George A Wells, Marco L A Sivilotti, Muhammad Mukarram, Brian H Rowe, Eddy Lang, Jeffrey J Perry, Robert Sheldon, Ian G Stiell, Monica Taljaard. Development of the Canadian Syncope Risk Score to predict serious adverse events after emergency department assessment of syncope. CMAJ. 2016;188(12):e289-e98.

4. Thiruganasambandamoorthy V, Sivilotti MLA, Le Sage N, Yan JW, Huang P, Hegdekar M, et al. Multicenter Emergency Department Validation of the Canadian Syncope Risk Score. JAMA Intern Med. 2020;180(5):737-44.
